# Rituximab impedes natural killer cell function in Chronic Fatigue Syndrome/Myalgic Encephalomyelitis patients: A pilot in vitro investigation

**DOI:** 10.1186/s40360-018-0203-8

**Published:** 2018-03-27

**Authors:** Natalie Eaton, Hélène Cabanas, Cassandra Balinas, Anne Klein, Donald Staines, Sonya Marshall-Gradisnik

**Affiliations:** 1 School of Medical Science, Griffith University, QLD, Gold Coast, Australia; 2 The National Centre for Neuroimmunology and Emerging Diseases, Menzies Health Institute Queensland, Griffith University, QLD, Gold Coast, Australia

**Keywords:** Chronic fatigue syndrome, Myalgic encephalomyelitis, Rituximab, Natural killer cells, Lytic proteins, Degranulation, Cytotoxicity

## Abstract

**Background:**

A recent in vitro pilot investigation reported Rituximab significantly reduced natural killer (NK) cell cytotoxicity in healthy donors. Chronic fatigue syndrome/Myalgic encephalomyelitis (CFS/ME) is a debilitating disorder of unknown etiology. A consistent finding is a significant reduction in NK cell cytotoxicity. Rituximab has been reported having questionable potential therapeutic benefits for the treatment of CFS/ME, however, the potential effects of Rituximab on NK cell cytotoxicity in CFS/ME patients are yet to be determined.

**Methods:**

A total of eight CFS/ME patients (48.63 ± 15.69 years) and nine non-fatigued controls (NFC) (37.56 ± 11.06 years) were included using the Fukuda case definition. Apoptotic function, lytic proteins and degranulation markers were measured on isolated NK cells using flow cytometry following overnight incubation with Rituximab at 10 μg/ml and 100 μg/ml.

**Results:**

There was a significant reduction in NK cell lysis between CFS/ME patients and NFC following incubation with Rituximab at 100 μg/ml at 12.5:1 and 6.25:1 effecter-target (E:T) ratios (*p* < 0.05). However, there was no significant difference for NFC following incubation with Rituximab at 10 μg/ml and 100 μg/ml.

There was no significant difference between CFS/ME patients and NFC for granzyme A and granzyme B prior to incubation with Rituximab and following overnight incubation with Rituximab at 10 μg/ml. There was a significant decrease in granzyme B in CFS/ME patients compared to NFC with 100 μg/ml of Rituximab prior to K562 cells stimulation (*p* < 0.05).

There was a significant increase in CD107a (*p* < 0.05) and CD107b expression (*p* < 0.01) in NFC after stimulation with K562 cells prior to incubation with Rituximab. There was a significant increase in CD107b expression between CFS/ME patients and NFC prior to incubation with Rituximab and without stimulation of K562 cells (*p* < 0.01). Importantly, there was a significant increase in CD107b following overnight incubation with 100 μg/ml of Rituximab in NFC prior to K562 cells stimulation (*p* < 0.01).

**Conclusion:**

This study reports significant decreases in NK cell lysis and a significant increase in NK cell degranulation following Rituximab incubation in vitro in CFS/ME patients, suggesting Rituximab may be toxic for NK cells. Caution should be observed in clinical trials until further investigations in a safe and controlled in vitro setting are completed.

**Electronic supplementary material:**

The online version of this article (10.1186/s40360-018-0203-8) contains supplementary material, which is available to authorized users.

## Background

CFS/ME is a debilitating disorder hallmarked by unexplained debilitating fatigue accompanied by immune, neurological, musculoskeletal, cardiovascular and gastrointestinal symptoms [1]. The diagnosis of CFS/ME is complex and relies on case definition for diagnostic criteria [1–3]. The underlying etiology of CFS/ME remains unknown; however, a significant reduction of NK cell cytotoxicity is a key and consistently reported feature of CFS/ME [4–14].

CFS/ME is believed to affect approximately 200,000 Australians [15] having a global prevalence of 0.2–6.4% [16]. CFS/ME is reported more commonly in women than in men, with 75% of patients being female [17] and predominantly affecting 30- to 40-year-olds in developed countries [16]. However, due to inconsistencies in CFS/ME case definitions the true prevalence is difficult to determine.

NK cells are effector lymphocytes of the innate immune system that eliminate pathogens and malignant cells, activate immune cells and provide cytokine producing functions [18]. NK cells have tightly regulated cytotoxic activity against stress and antibody-coated cells [18–22]. The majority of human peripheral NK cells are CD56^dim^ NK cell subset bearing the low-affinity Fc-γ-receptor CD16 [23]. CD16 binds to the Fc portion of immunoglobulin (Ig) G and mediates antibody-dependent cellular cytotoxicity (ADCC) [23–26]. NK cell cytotoxicity (NKCC) involves numerous steps including adhesion to the target cell, activations of surface receptors, polarization of secretory granules and release of lytic proteins, including granzyme A and granzyme B, to induce apoptosis of the target cell [7, 21, 27].

NK cell activation is tightly regulated by activating receptors that recognise pathogen-derived, stress-induced and tumour specific ligands [20]. CD16 plays a prominent role as an activating receptor for NK cells [28]. NK cell activation initiates calcium (Ca^2+^)-dependent signal transduction through receptor cytoplasmic tails that contain immunoreceptor tyrosine-based activation motifs (ITAM) [11, 22, 29–31]. Ligation of ITAM-bearing receptor complexes results in the recruitment and activation of mitogen-activated protein kinase (MAPK) phosphorylation cascade [29]. The phosphorylation of kinases induces the polarization of NK cell granules via the microtubule-organizing centre (MTOC) [32, 33]. Granule polarization ensures granule contents are released facing the target cell. NK cell cytotoxic granules are responsible for the storage and secretion of lytic proteins including perforin, a membrane-disrupting protein, and granzymes, a family of proteases [11, 27]. The secretion of perforin is suggested to create pores within the target cell membrane to facilitate endocytosis mechanisms, in which granzymes can enter the target cell to trigger apoptosis by cleaving pro-apoptotic caspases and influence nuclear damage [5, 7, 27, 34, 35].

Importantly, reduced NK cell function is the most consistently reported finding in both severe and moderate CFS/ME patients [4–14]. Impaired NKCC in CFS/ME patients is evident through delayed degranulation [7, 36, 37] and decreased lytic proteins, predominantly granzyme B [5, 11, 36, 38, 39]. The increase in NK cell degranulation in CFS/ME patients may suggest an inability to induce sufficient cytotoxic lysis or continued activation due to insufficient lytic proteins. Therefore, using flow cytometry to investigate expression of degranulation markers, CD107a and CD107b, and intracellular lytic proteins, granzyme A and granzyme B, is critical when investigating the cytotoxic activity of NK cells [40].

Rituximab (RTX), is a chimeric antibody that targets CD20 present on healthy and malignant B lymphocytes. RTX can trigger target cell death through three effector functions: 1) programmed cell death, 2) induction of complement-mediated cytotoxicity, and 3) ADCC mediated by Fc receptor-bearing immune cells such as NK cells [41]. RTX works to opsonize the CD20 surface marker on B lymphocytes, this stimulates the recruitment of NK cells and ligation with CD16. The activation of CD16 to the Fc portion of RTX activates Ca^2+^-dependent elimination of B lymphocytes through ADCC.

Limited investigations have examined the potential role of therapeutic interventions in CFS/ME patients. We and others have reported elevated CD20^+^ B lymphocytes in CFS/ME patients [9, 42–44]. Moreover other investigators have employed anti-CD20 therapy as a possible therapeutic approach for the treatment of CFS/ME [45–47], where CFS/ME patients received two infusions at 500 mg/m^2^ of RTX two weeks apart. Clinical improvement was self-reported in sixty-four and 67 % of participants in these two separate studies [45, 47]; however this improvement was only maintained in 26.6% of patients twelve months post administration.

Importantly, a recent study conducted by Merkt et al. reported that in vitro treatment of NK cells with RTX at a concentration of 10 μg/ml resulted in significant inhibition of NK cell cytotoxic activity from healthy control donors [41]. This investigation also reported a significant reduction of lytic proteins, predominantly granzyme B, and phenotypical and functional changes to CD16 [41] in NK cells following RTX incubation from non-fatigued controls donors.

The effect of RTX on NK cells in malignancies and rheumatic diseases is documented [41, 48–50]. However, the possible role of RTX modulating NK cell cytotoxicity in CFS/ME patients is unknown as Lunde et al. only investigated NK cell subset numbers in CFS/ME patients receiving 500 mg/m^2^ of RTX [51]. Therefore, this investigation aimed to examine NK cell cytotoxic activity, lytic proteins and degranulation following incubation of RTX at varying concentrations with NK cells from CFS/ME patients in a controlled and safe laboratory setting in vitro.

## Methods

### Study Participants

Participants were sourced from the National Centre for Neuroimmunology and Emerging Diseases (NCNED) research database for CFS/ME. Participants were aged between 18 and 65 years, and recruited from South East Queensland and Northern on New South Wales where ME/CFS patients were defined by the 1994 Center for Disease Control and Prevention criteria for CFS/ME. NFC were healthy volunteers that reported no incidence of CFS/ME or fatigue and were in good health without evidence of illness or co-morbidities. Participants were screening according to routine pathology tests and completed a comprehensive questionnaire corresponding with the International Consensus Criteria (ICC) [1]. Participants were excluded from this study if history of smoking, alcohol abuse, autoimmune diseases, cardiac disease, diabetes or co-morbidities were reported.

Six of the eight CFS/ME patients documented minor interventions aimed to control symptoms including cognitive impairment, sleep disturbances, periodic gastro-intestinal symptoms and pain. No participants reported chronic immunosuppressant therapy, immunomodulators or any current medications that may potentially affect the immunological findings reported in the results of this pilot investigation.

### Peripheral Blood Mononuclear Cells Isolation and Natural Killer Cells Isolation

Participants donated 85 ml of whole blood which was collected in ethylendiaminetetraacetic acid (EDTA) tubes between 8:00 am and 11:00 am. Routine full blood analysis was analyzed from 5 ml of blood within 4 h of collection for red blood cell counts, lymphocytes and granulocytes using an automated cell counter (ACT differential analyser; Beckman Coulter, Brea, CA, USA) (Table 1). Participants were screened and excluded if blood parameters were outside the normal range.

**Table 1:** Blood parameters and patient demographic measured in CFS/ME patients and NFC groups

	NFC (*n* = 9)	CFS/ME (*n* = 8)	*P* value
Age (years)	37.56 ± 11.06	48.63 ± 15.69	0.503
Gender			
Male *n(%)*	3(33.3)	1 (12.5)	
Female *n(%)*	6 (66.7)	7 (87.5)	
Pathology			
White Cell Count (× 10^9^/L)	5.94 ± 1.61	5.45 ± 1.09	0.664
Neutrophils (×10^9^/L)	3.54 ± 1.49	3.36 ± 0.82	0.847
Lymphocytes (× 10^9^/L)	1.87 ± 0.38	1.70 ± 0.49	0.248
Monocytes (× 10^9^/L)	0.32 ± 0.11	0.32 ± 0.06	0.962
Eosinophils (× 10^9^/L)	0.18 ± 0.09	0.18 ± 0.12	0.885
Platelet (× 10^9^/L)	240.78 ± 47.65	258.88 ± 37.13	0.413
Haemoglobin (g/L)	132.67 ± 8.51	135.38 ± 7.82	0.384
Haematocrit	0.37 ± 0.10	0.42 ± 0.03	0.191
Red Cell Count (× 10^12^/L)	4.53 ± 0.34	4.49 ± 0.34	0.923
MCV fl	89.67 ± 2.74	92.88 ± 5.11	0.222

Results from white and red blood cell parameters measured in CFS/ME and control groups. Comparisons of blood parameters between the CFS/ME and NFC revealed no significant differences. No significant differences were observed in patient age distribution. Data presented as mean ± standard deviation. *Abbreviations: NFC, non-fatigued controls; CFS/ME, chronic fatigue syndrome; ME, Myalgic encephalomyelitis*

Peripheral blood mononuclear cells (PBMCs) were isolated from 80 ml of whole blood by centrifugation over a density gradient medium (Ficoll-Paque Premium; GE Healthcare, Uppsala, Sweden). PBMCs were stained with trypan blue (Invitrogen, Carlsbad, CA) to determine cell count and cell viability, and adjusted to a final concentration of 5 × 10^7^ cells/ml.

**Fig. 1:**
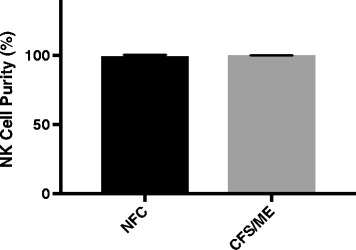
Natural Killer cell purity. Bar graphs representing isolated NK cell purity for NFC and CFS/ME patients. Data presented as mean ± standard deviation. HC = 99.46 ± 0.904 CFS/ME = 99.85 ± 0.233. *Abbreviations: NFC, non-fatigued controls; CFS, chronic fatigue syndrome; ME, myalgic encephalomyelitis; NK, natural killer*

NK cells were isolated from PBMCs using an EasySep Negative Human NK Cell Isolation Kit (Stem Cell Technologies, Vancouver, BC, Canada). NK cells purity was measured after staining with CD56 (0.25 μg/5 μl) and CD3 (0.25 μg/20 μl) antibodies for 20 min at room temperature and analyzed using a LSR-Fortessa X20 flow cytometer (Becton Dickinson [BD] Biosciences, San Diego, CA, USA). NK cells purity was 99.85% ± 0.24% and 99.46% ± 0.91% for CFS/ME and NFC, respectfully (Fig. 1).

### Rituximab Treatment

NK cells were treated with concentrations of 10 μg/ml as previously reported [41] and 100 μg/ml of RTX (Rituxan, Genentech, CA, USA) and incubated for 24 h at 37 °C with 5% CO_2_ in RPMI-1640 (Invitrogen Life Technologies, Carlsbad, CA, USA) supplemented with 10% fetal bovine serum (FBS) (Invitrogen Life Technologies, Carlsbad, CA, USA).

### Natural Killer Cell Cytotoxicity Assay

NK cytotoxic activity was conducted as previously described [52]. NK cells were labelled with Paul Karl Horan (PKH-26) (3.5 μl/test) (2 × 10^− 6^ M) for 5 min (Sigma-Aldrich, St. Louis. MO, USA) and incubated with K562 cells for 4 h at 37 °C with 5% CO_2_ in RPMI-1640 supplemented with 10% FBS. The concentration of NK cells and K562 cells were adjusted to 5 × 10^5^ cells/ml and 1 × 10^6^ cells/ml, respectively. During incubation, cells were combined at E:T ratios of 12.5:1 and 6.25:1 in addition to controlling samples. Control and RTX treated cells were stained using Annexin V (2.5 μl/test) (BD Bioscience, San Jose, CA, USA) and 7-amino-actinomycin (7-AAD) (2.5 μl/test) (BD Bioscience, San Jose, CA, USA) to determine apoptosis using flow cytometry analysis recording 10,000 events (Additional file 1). E:T ratio of 12.5: 1, 6.25 and control were used to assess cytotoxic activity [38, 40, 53]. NK cytotoxic activity was calculated as percent specific death of the K562 cells for the two E:T ratios as previously described [40, 52]. The percentage of target cell lysis was calculated from:

$$ Cytotoxicity\ \left(\%\right)=\frac{\left( early\ stage\ apoptosis+ late\ stage\ apoptosis+ dead\ K562\  cells\right)}{All\ K562\  Cells}\times 100 $$

### Natural Killer Cell Lytic Proteins Assay

Intracellular staining was used to measure granzyme A and granzyme B as previously described [7]. NK cells and K562 cells were plated and placed in the incubator at 37 °C for 4 h with 5% CO_2_ at E:T 25:1 in RPMI-1640 supplemented with 10% FBS. NK cells were incubated with Monensin (0.67 μl/ml) and Brefeldin A (1 μl/ml) (BD Bioscience, San Jose, CA, USA) to interfere with degranulation and intracellular cytokine transport from the endoplasmic reticulum. NK cells were stained with monoclonal antibodies for granzyme A FITC (0.5 μg/20 μl) and granzyme B BV450 (0.125 μg/5 μl) (BD Bioscience, San Jose, CA, USA) for 30 min at 4 °C. Both control and treated NK cells were measured using flow cytometric analysis recording 10,000 events (Additional file 1).

### Natural Killer Cell Degranulation Assay

NK cell surface expression of CD107a and CD107b was measured as a marker for NK cell degranulation and determined as previously described [54]. NK cell concentration was adjusted to 1 × 10^5^ cells/ml and incubated in the presence of CD107a APC (0.06 μg/5 μl) and CD107b FITC (1 μg/20 μl) (BD Bioscience, San Jose, CA, USA) and stimulated by K562 cells (1 × 10^4^ cells/ml) for 4 h at 37 °C with 5% CO_2_ at E:T 25:1 in RPMI-1640 supplemented with 10% FBS. NK cells required the addition of monensin (0.67 μl/ml) to prevent degranulation and Brefeldin A (1 μl/ml) to block exocytosis of cytokines. Both control and treated cells were measured using flow cytometric analysis recording 10,000 events.

### Statistical Analysis

Pilot data from this investigation were analyzed using SPSS version 24 (IBM Corp, Version 24, Armonk, NY, USA) and GraphPad Prism, version 7 (GraphPad Software Inc., Version 7, La Jolla, CA, USA). All data sets were tested for normality using the Shapiro–Wilk test. The independent Mann–Whitney U test was used to identify any significant differences in the NK cell parameters between the CFS/ME and NFC groups. Significance was set at *p* < 0.05 and the data are presented as mean ± standard deviation unless otherwise stated.

## Results

### Participant characteristics, blood parameters and NK cell purity

There were eight CFS patients (48.63 ± 15.69 years), of which seven were females, who met the 1994 Fukuda definition (mean age [years] ± Standard deviation [SD]). There were nine NFC (37.56 ± 11.06 years), of which six were females. There were no significant differences between groups for age (Table 1). All participants in both groups were of European descent and were residents of Australia at the time of blood collection. Comparison of group ages and blood parameters between CFS/ME patients and NFC found no significant differences (Table 1). Isolated NK cell purity was determined using flow cytometry, where NK cell purity for CFS/ME patients was 99.85 ± 0.233 and NFC was 99.46 ± 0.904 (Fig. 1).

### Rituximab leads to significant difference in cytotoxic activity of NK cells

**Fig. 2:**
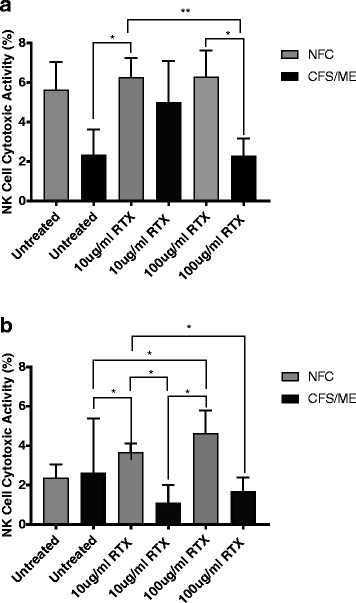
Natural killer cell cytotoxicity. Bar graph plots representing in vitro assessment of NK (cytotoxic activity) of tumour-cell lines K562 in CFS/ME (*n* = 8) and NFC (*n* = 9). Lytic activity represented by percentage lysis of target cells on the y-axis. **(a).** Represents data at 12.5:1 effector target ratio. **(b).** Represents data at 6.25:1 effector target ratio. Data presented as mean ± standard deviation. * refers to significant difference where *p* < 0.05 and ** refers to significant difference where *p* < 0.01. *Abbreviations: NK, natural killer; ME, myalgic encephalomyelitis; CFS, chronic fatigue syndrome; NFC, non-fatigued control; RTX, Rituximab; NK, natural killer*

Cytotoxic activity was determined using flow cytometry to assess NK cell lysis of the tumour target K562 cell line for both NFC (*n* = 9) and CFS/ME patients (*n* = 8). There was a significant reduction in NK lysis in CFS/ME patients compared to NFC following incubation with RTX at 100 μg/ml (Fig. 2a) (*p* < 0.05) and NK lysis in CFS/ME patients compared to NFC following RTX at 10 μg/ml (Fig. 2b) (p < 0.05). There was no significant difference in NK cell lysis for NFC following overnight incubation with RTX at 10 μg/ml and 100 μg/ml (Fig. 2a).

### Rituximab leads to significant decrease in NK cell lytic protein granzyme B

**Fig. 3:**
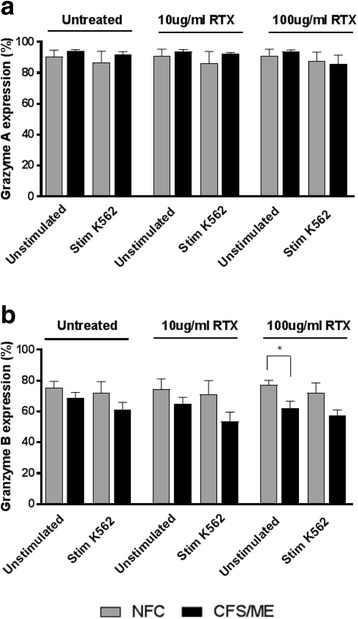
Natural killer cell lytic proteins. Bar graph plots representing in vitro assessment of NK cell lytic proteins. Data is represented as either unstimulated or stimulated with K562 target cells, and treated or untreated with RTX. **(a)** granzyme A and **(b)** granzyme B. Data is represented as the percentage of intracellular lytic proteins on the y-axis. Data presented as mean ± standard deviation. * refers to significant difference where *p* < 0.05. *Abbreviations: CFS, chronic fatigue syndrome; Stim, stimulated; RTX, Rituximab; NFC, non-fatigued controls; ME, myalgic encephalomyelitis*

Of the nine NFC, four of these were selected for lytic protein analysis due to concentration of NK cells following isolation. There was no significant difference between groups for lytic proteins for NFC (*n* = 4) and CFS/ME patients (n = 8) at baseline prior to overnight incubation with RTX for granzyme A (Fig. 3a) and granzyme B (Fig. 3b). No significant difference was shown for lytic proteins following overnight incubation with RTX at 10 μg/ml as well as for granzyme A following overnight incubation with 100 μg/ml of RTX (Fig. 3a). Importantly, there was a significant decrease in granzyme B between NFC and CFS/ME patients incubated with 100 μg/ml of RTX prior to stimulation using K562 cells, while there was no significant change following K562 cell stimulation (Fig. 3b).

### Rituximab leads to a significant increase in Natural Killer cell degranulation

**Fig. 4:**
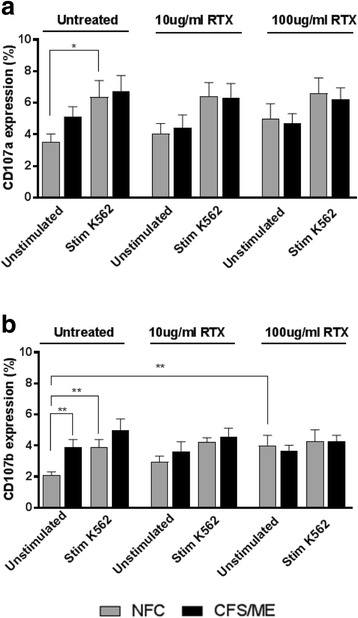
Natural killer cell degranulation. Bar graph plots representing in vitro assessment of NK cell degranulation markers. Data is represented as either unstimulated or stimulated with K562 target cells, and treated or untreated with RTX. **a** CD107a and **b** CD107b in CFS/ME (*n* = 6) and NFC (*n* = 7). Data is represented as the percentage of extracellular degranulation markers. Data presented as mean ± standard deviation. * refers to significant difference where p < 0.05 and ** refers to significance difference where p < 0.01. *Abbreviations: CFS, chronic fatigue syndrome; NFC, non-fatigued controls; RTX, rituximab; Stim, stimulated; ME, myalgic encephalomyelitis*

Of the nine NFC, six participants were used for degranulation due to the concentration of NK cells following isolation. There was no significant difference between groups for degranulation for NFC (*n* = 6) and CFS/ME patients (*n* = 7) at baseline prior to overnight incubation with RTX for CD107a (Fig. 4a). There was a significant difference in CD107a expression in NFC when stimulated with K562 cell line prior to overnight incubation with RTX (*p* < 0.05). There was an increase in degranulation observed in NFC prior to K562 cell stimulation following overnight incubation with RTX approaching statistical significance.

Similarly, there was a significant difference observed within and between groups for CD107b expression prior to incubation with RTX (Fig. 4b). Importantly, there was an increase in CD107b expression between NFC after stimulation with K562 cells. There was a significant increase in CD107b expression in CFS/ME patients in comparison to NFC at baseline prior to incubation with RTX and without stimulation of K562 cells (*p* < 0.01). Importantly, there was a significance increase in CD107b following overnight incubation with 100 μg/ml of RTX observed in NFC prior to K562 cell stimulation (*p* < 0.01).

## Discussion

This is the first study to investigate NK cell cytotoxic activity following in vitro incubation with RTX at 10 μg/ml and 100 μg/ml in CFS/ME patients and NFC. The present study examined cytotoxic and apoptotic mechanisms through lysis of target K562 cells, intracellular staining of lytic proteins including granzyme A and granzyme B, and extracellular straining of degranulation markers, CD107a and CD107b.

We report novel findings as there were significant reductions in NK cell lysis in CFS/ME patients compared with NFC following incubation with RTX at 100 μg/ml at 12.5:1 E:T (*p* < 0.05). Significance was also observed between groups at 6.25:1 E:T for 10 μg/ml of RTX. A decrease was observed in CFS/ME patients compared with NFC at 100 μg/ml of RTX, however this was not significant possibly due to the small sample size. Previous in vitro investigations using RTX at different concentrations ranging from 10 μg/ml to 200 μg/ml have reported reduced cytotoxic activity of NK cells isolated from healthy donors, chronic lymphocytic leukaemia (CLL) patients and non-Hodgkin lymphoma patients [41, 48, 50]. The significant decrease in cytotoxic activity observed in this study suggests that in vitro RTX at 100 μg/ml impedes NK cell function. Studies completed by us and other researchers have reported significantly reduced NKCC in CFS/ME patients [4–14]. Therefore, RTX may have adverse outcomes for CFS/ME patients as reduced NK cell function impairs the ability to clear virus-infected and malignant cells.

This current investigation did not report significant difference in NFC for NK cell lysis of target K562 cells. In contrast, Merkt et al. reported significant decrease in NK cell cytotoxic activity of healthy donors (*n* = 3) [41]. Additionally, Capuano et al. reported defective NK cell cytotoxic response in healthy donors and CLL patients [50]. However, these studies are not without limitations with the low sample numbers and limited screening definition of healthy control. Without clear indication of criteria for healthy control inclusion in these studies, it can be assumed that these participants may be susceptible to reduced NK cell function. The NFC participating in our current investigation are included using a detailed questionnaire derived from the Fukuda, Canadian Consensus Criteria and ICC [1–3]. Additionally, investigations by Merkt et al. and Capuano et al. used the chromium (Cr)-51 release assay. The ^51^Cr-release assay is used to measure cytotoxic activity, however, comparisons with flow cytometric analysis have shown cytometric methods to be more sensitive with higher lysis generated [52, 55].

Lytic proteins are apoptotic-inducing proteins released from cytotoxic granules in NK cells. Intracellular staining was used to investigate granzyme A and granzyme B using flow cytometric analysis. We report a significant decrease in granzyme B in CFS/ME patients compared with NFC following incubation with 100 μg/ml of RTX prior to stimulation using K562 target cells (*p* < 0.05). Previous work performed by our group and other researchers have reported a significant reduction in granzyme B in NK cells isolated from CFS/ME patients when stimulated with K562 target cells [5, 36, 38, 39]. Granzyme B is responsible for apoptosis activation by cleaving substrates at aspartic acid leading to caspase-3 activation [56]. Importantly, the decrease in granzyme B following incubation with RTX at 100 μg/ml prior to NK cell stimulating using K562 cells demonstrates that RTX can influence cytolytic activity of NK cells.

Previous research has reported that lytic proteins negatively correlate with degranulation, suggesting a decrease in lytic proteins is associated with an increase in degranulation markers [40]. Degranulation is a critical process by which cytotoxic granules polarize towards the immune synapse to release lytic proteins towards the target cell [27]. Extracellular staining was used to investigate degranulation markers CD107a and CD107b using flow cytometric analysis. We report a significant difference in CD107a expression in NFC when stimulated with K562 cells prior to overnight incubation with RTX (*p* < 0.05) and conversely a significant increase in CD107b expression was found in NFC after stimulation with K562 target cells (*p* < 0.01). Importantly, we reported for the first time a significant increase in CD107b expression in CFS/ME patients in comparison to NFC prior to incubation with RTX and without stimulation of K562 cells (*p* < 0.05). Previous research has reported significant increase in CD107a in NK cells isolated from CFS/ME patients compared with NFC following stimulation with K562 cells [36, 37, 53]. Huth et al. suggested that the increase in degranulation markers in CFS/ME patients suggests continued activation due to inability to induce sufficient cytotoxic lysis of target cells [53].

Finally, we also report novel findings where a significant increase in CD107b expression following overnight incubation with 100 μg/ml of RTX was observed in NFC prior to K562 cell stimulation (*p* < 0.01). Therefore, this suggests that RTX may stimulate Ca^2+^ influx required for NK cell degranulation. Additionally, as there were no significant changes in CD107a following overnight incubation with RTX at 10 μg/ml and 100 μg/ml, our results are not consistent with previous research completed by Merkt et al. who reported an increase in degranulation in healthy donors (*n* = 6) treated with 10 μg/ml of RTX [41]. However, limited information was provided regarding the methodology of degranulation assays in that study. Additional in vitro studies using RTX have shown that continued NK cell activation causes CD16 down regulation, therefore impairing NK cell cytotoxic activity [41, 48, 49]. As there were no significant changes for CFS/ME patients when incubated overnight with RTX at 10 μg/ml for CD107a and CD107b, and CD107a with RTX at 10 μg/ml, this suggests that RTX does not improve NK cell degranulation. Further research is needed to determine whether RTX changes intracellular signalling of MAPK and ERK1/2 phosphorylation which is reported to be delayed in CFS/ME patients [53].

Our results demonstrate that RTX may have no benefit for the treatment of CFS/ME patients. Additionally, there are limited pharmacokinetic investigations for the standard dose of RTX for the treatment of CFS/ME. The standard dose of RTX is 375 mg/m^2^ two weeks apart for non-Hodgkin Lymphoma patients [57, 58]. Herlt et al. suggests that patients treated for autoimmune diseases require a lower dose of RTX as a single infusion with 100 mg/m^2^ effectively induces B lymphocyte depletion [58]. For the phase II clinical trial on CFS/ME patients by Fluge et al. RTX infusions were administered to patients at 500 mg/m^2^ two weeks apart. Additional maintenance infusions were given after 3, 6, 10 and 15 months with two patients receiving as high as 11 doses, which suggests that CFS/ME patients receive only short term therapeutic effects from RTX treatment [47]. Given the literature in CFS/ME and autoimmunity is inconsistent and inconclusive, further investigations are required to understand the immunological and pharmaceutical mechanisms of RTX in non-malignant diseases.

The novel, preliminary findings of our study provide a rationale for further investigations into a larger cohort to investigate the possible therapeutic role of RTX on NK cell function, notably NK cytotoxicity. The results from this study highlight the importance of in vitro investigations during clinical trials. Furthermore, the reduction in NK cell lysis between NFC and CFS/ME patients with 100 μg/ml of RTX may indicate that RTX impedes NKCC. Further investigation is required for NK cell phenotypes and for CD16 polymorphisms due to their role in efficacy of RTX therapy as high-affinity polymorphisms correlate with better clinical responses to RTX [49].

This current investigation highlights the possible role of RTX in vitro and may not necessarily recreate in vivo conditions. Two ex vivo investigations reported that RTX significantly increased NK cell degranulation [59] and reduced CD16-dependent NKCC [60] in lymphoma patients. Cox et al. suggested that downregulation CD16 impairs RTX recognition that facilitates NKCC. Moreover, the decision to incubate isolated NK cells for 24 h with RTX is supported by previous investigators [41]. The current findings from this investigation are supported by previous research where prolonged treatment in vivo and in vitro with RTX leads to functional impairment of NK cells [41, 50, 59, 60]. Therefore, high doses of RTX may impair NK cell functionality.

## Conclusion

This investigation, using isolated NK cells and flow cytometry, is novel as it is the first to investigate the effects of RTX on the NK cells of CFS/ME patients. The results of this study demonstrate significant differences between NFC and CFS/ME patients following overnight incubation with 100 μg/ml of RTX. Our results suggest that RTX may have toxicological effects on NK cells isolated from CFS/ME patients. Further in vitro investigations aimed at examining the effect of RTX on isolated NK cell cytotoxic function in CFS/ME patients are warranted.

## Additional File

Additional file1: **Figure S1.** Representative flow cytometric plot of NK cell cytotoxic activity. PKH-26 was used to gate NK cells. PKH-26 negative cells were identified as K562 target cells and used to determine apoptosis. Annexin V was used to determine events undergoing apoptosis and 7-AAD was used to determine cells undergoing late apoptosis. **Figure S2.** Representative flow cytometric plot of intracellular staining analysis of lytic proteins. NK cells were isolated and were used is isolation purity was ≥95%. NK cells were selectively gated on using flow cytometry and isotype controls were used to determine the positive population for Granzyme A and Granzyme B expression. **Figure S3.** Representative flow cytometry plots for degranulation markers. NK cells were isolated and were used if isolation purity was ≥95%. NK cells were gated and selected using flow cytometry to determine CD107a and CD107b expression. Isotype controls were used to determine the positive population. (PDF 231 kb)

## Abbreviations

7-AAD: 7-amino-actinomycin
BD: Becton Dickinson
Ca^2+^: Calcium
CFS: Chronic fatigue syndrome
E:T: Effecter-target
EDTA: ethylendiaminetetraacetic acid
FBS: Fetal bovine serum
ICC: International Consensus Criteria
Ig: Immunoglobulin
ITAM: Immunoreceptor tyrosine-based activation motif
MAPK: Mitogen-activated protein kinase
ME: Myalgic Encephalomyelitis.
MTOC: Microtubule-organising centre.
NCNED: National Centre for Neuroimmunology and Emerging Diseases.
NFC: Non-fatigued controls.
NK: Natural killer.
NKCC: Natural killer cell cytotoxicity.
PBMC: Peripheral blood mononuclear cells.
PKH: Paul Karl Horan.
RTX: Rituximab.
