# Supplementary material for: Rituximab impedes natural killer cell function in Chronic Fatigue Syndrome/Myalgic Encephalomyelitis patients: A pilot in vitro investigation
Source: BMC Pharmacol Toxicol. 2018 Mar 27;19:12. doi: 10.1186/s40360-018-0203-8 (PMC5870391; doi:10.1186/s40360-018-0203-8)
Supplement: Supplementary file 1 — Figure S1. Representative flow cytometric plot of NK cell cytotoxic activity. PKH-26 was used to gate NK cells. PKH-26 negative cells were identified as K562 target cells and used to determine apoptosis. Annexin V was used to determine events undergoing apoptosis and 7-AAD was used to determine cells undergoing late apoptosis. Figure S2. Representative flow cytometric plot of intracellular staining analysis of lytic proteins. NK cells were isolated and were used is isolation purity was ≥95%. NK cells were selectively gated on using flow cytometry and isotype controls were used to determine the positive population for Granzyme A and Granzyme B expression. Figure S3. Representative flow cytometry plots for degranulation markers. NK cells were isolated and were used if isolation purity was ≥95%. NK cells were gated and selected using flow cytometry to determine CD107a and CD107b expression. Isotype controls were used to determine the positive population. (PDF 231 kb) [file 40360_2018_203_MOESM1_ESM.pdf]

Supplementary Figure 1:

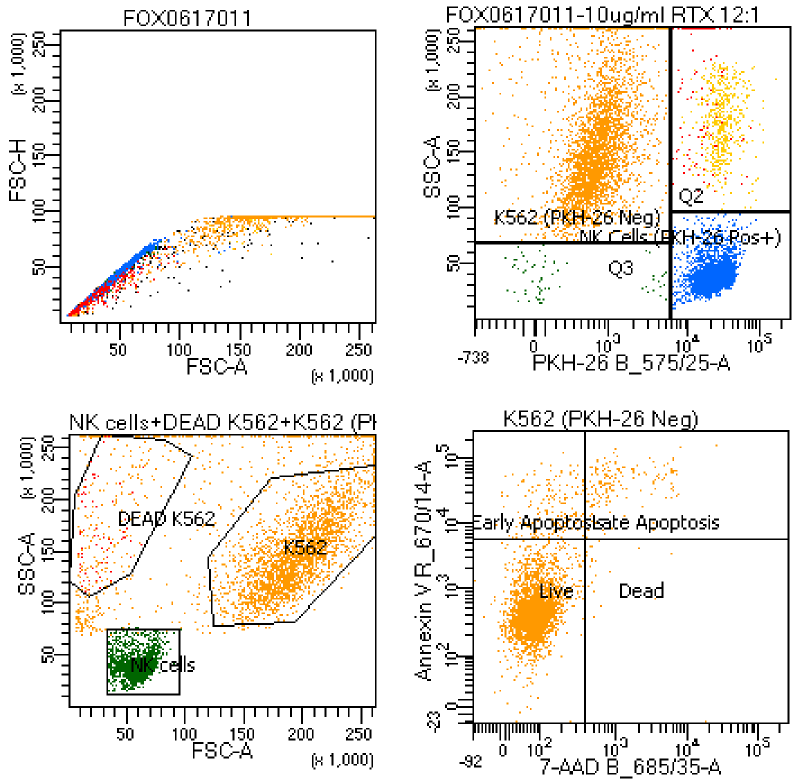

Tube: 10ug/ml RTX 12:1

Population	#Events	%Parent	%Total
All Events	10,000	####	100.0
K562	3,132	31.3	31.3
NK cells	4,473	44.7	44.7
K562 (PKH-26 Neg)	3,335	33.4	33.4
Early Apoptosis	177	5.3	1.8
Late Apoptosis	177	5.3	1.8
Live	2,957	88.7	29.6
Dead	24	0.7	0.2
Q1-1	0	0.0	0.0
Q2-1	0	0.0	0.0
Q3-1	18	75.0	0.2
Q4-1	6	25.0	0.1
Q2	1,187	11.9	11.9
Q3	691	6.9	6.9
NK Cells (PKH-26 Pos+)	4,787	47.9	47.9
DEAD K562	283	2.8	2.8

Supplementary Figure 1: Representative flow cytometric plot of NK cell cytotoxic activity. PKH-26 was used to gate NK cells. PKH-26 negative cells were identified as K562 cells and used to determine apoptosis. Annexin V was used to determine events undergoing apoptosis and 7-AAD was used to determine cells undergoing late apoptosis and cells that were dead.

Supplementary Figure 2:

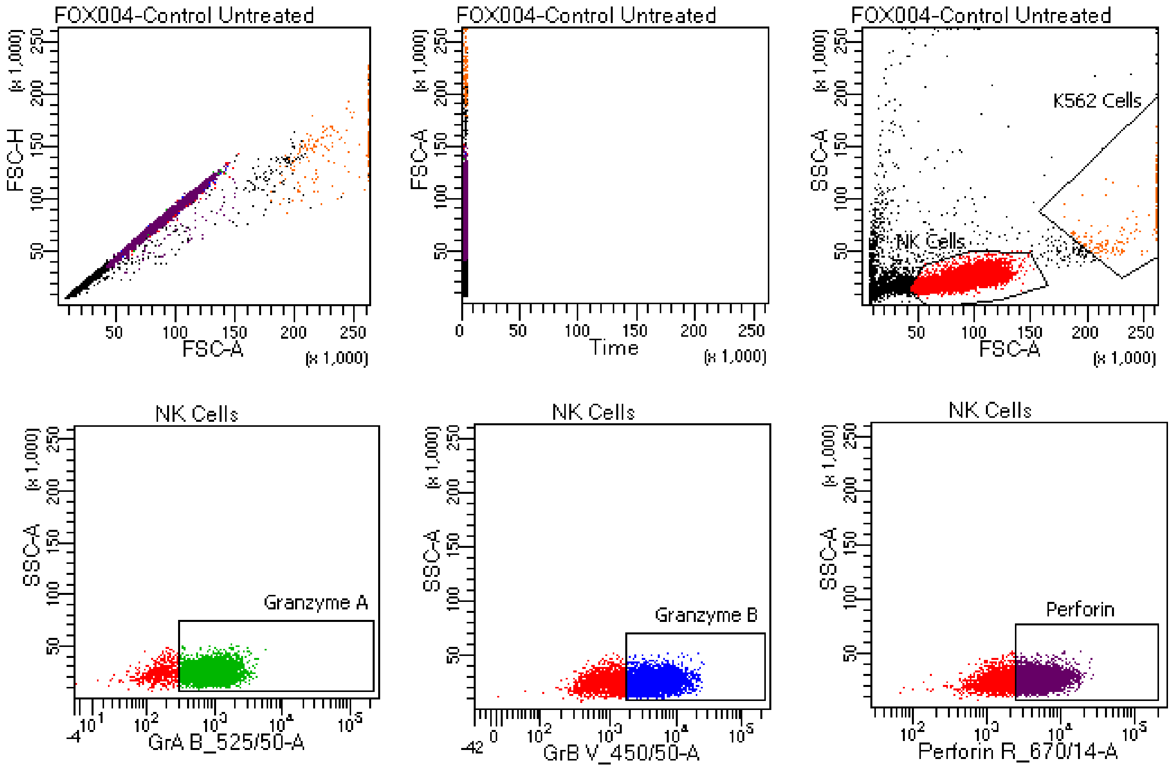

Experiment Name:	NK Lytic Proteins_001
Specimen Name:	FOX004
Tube Name:	Control Untreated
Record Date:	Jun 8, 2017 7:36:00 PM
$OP:	Natalie

Population	#Events	%Parent	%Total	GrA B_525/5... Median	Perforin R_67... Median	GrB V_450/5... Median
NK Cells	7,951	79.5	79.5	858	3,671	3,280
K562 Cells	188	1.9	1.9	2,547	11,300	9,992
Granzyme A	7,353	92.5	73.5	903	3,905	3,542
Granzyme B	5,927	74.5	59.3	958	4,360	4,249
Perforin	5,820	73.2	58.2	936	4,553	4,060

Supplementary Figure 2: Representative flow cytometric plot of intracellular staining analysis of lytic proteins. NK cells were isolated and were used if isolation purity was ≥95%. NK cells were selectively gated on using flow cytometry and isotype controls were used to determine the positive population for Granzyme A and Granzyme B expression.

Supplementary Figure 3:

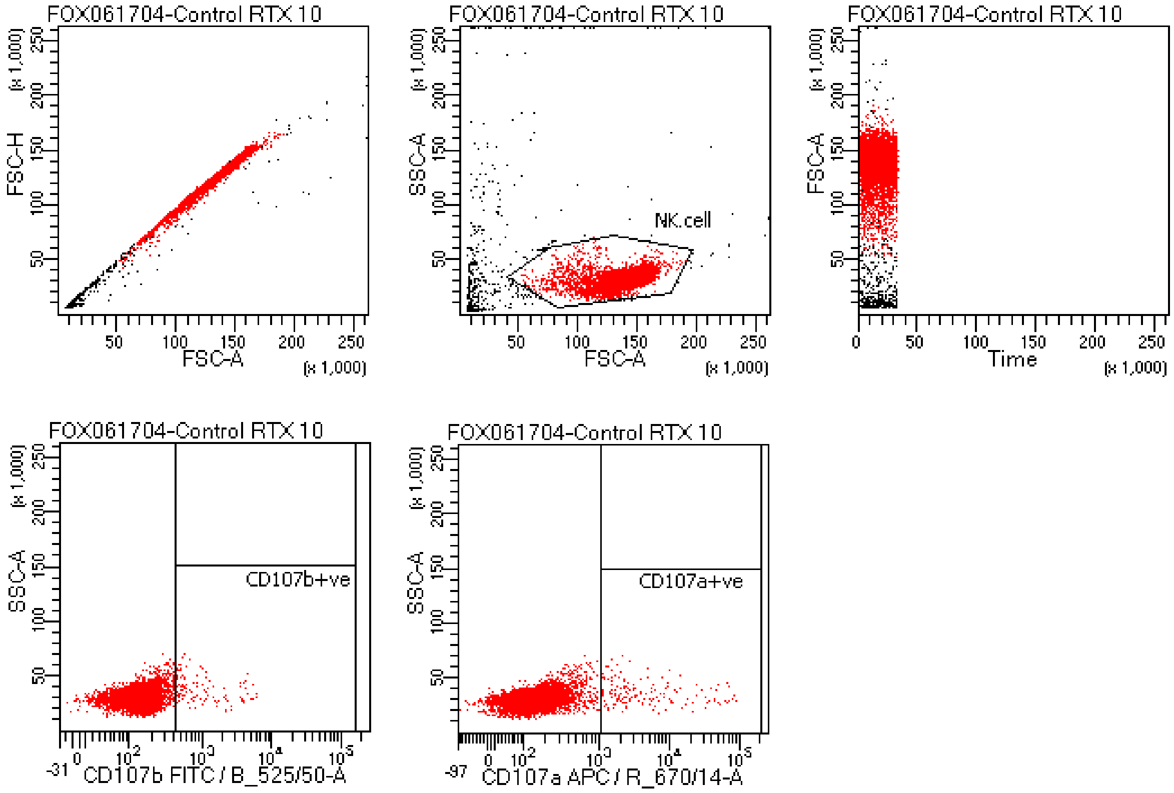

Experiment Name:	NK Cell Degranulation_001
Specimen Name:	FOX061704
Tube Name:	Control RTX 10
Record Date:	Jun 8, 2017 6:10:51 PM
$OP:	Natalie

Population	#Events	%Parent	%Total	CD107b FITC / B... Median	CD107a APC / R... Median
All Events	10,130	####	100.0	131	169
NK cell	9,648	95.2	95.2	131	167
CD107b+ve	112	1.2	1.1	733	5,064
CD107a+ve	148	1.5	1.5	511	2,925

Tube: Control RTX 10

Population	#Events	%Parent	%Total
All Events	10,130	####	100.0
NK cell	9,648	95.2	95.2
CD107b+ve	112	1.2	1.1
CD107a+ve	148	1.5	1.5

Supplementary Figure 3: **Representative flow cytometric plots for degranulation markers.** NK cells were isolated and were used if isolation purity was ≥95%. NK cells were gated and selected using flow cytometry to determine CD107a and CD107b expression. Isotype controls were used to determine the positive population.
